# Symbol/Meaning Paired-Associate Recall: An “Archetypal Memory” Advantage?

**DOI:** 10.3390/bs3040541

**Published:** 2013-10-09

**Authors:** Milena Sotirova-Kohli, Klaus Opwis, Christian Roesler, Steven M. Smith, David H. Rosen, Jyotsna Vaid, Valentin Djonov

**Affiliations:** 1 Department of Psychology, University of Basel, Missionstrasse60/62, Basel 4055, Switzerland; E-Mails: klaus.opwis@unibas.ch (K.O.); christian.roesler@kh-freiburg.de (C.R.); 2 Department of Psychology, Texas A&M University, College Station, TX 77843, USA; E-Mails: stevesmith@tamu.edu (S.M.S.); jvaid@tamu.edu (J.V.); 3 School of Medicine, Oregon Health and Science University, Portland, OR 97239, USA; E-Mail: drdavidhrosen@gmail.com; 4 Institute of Anatomy, University of Bern, Balzerstrasse 2, Bern 3000, Switzerland; E-Mail: valentin.djonov@ana.unibe.ch

**Keywords:** archetypes, collective unconscious, memory, cross-cultural study

## Abstract

The theory of the archetypes and the hypothesis of the collective unconscious are two of the central characteristics of analytical psychology. These provoke, however, varying reactions among academic psychologists. Empirical studies which test these hypotheses are rare. Rosen, Smith, Huston and Gonzales proposed a cognitive psychological experimental paradigm to investigate the nature of archetypes and the collective unconscious as archetypal (evolutionary) memory. In this article we report the results of a cross-cultural replication of Rosen *et al.* conducted in the German-speaking part of Switzerland. In short, this experiment corroborated previous findings by Rosen *et al.*, based on English speakers, and demonstrated a recall advantage for archetypal symbol meaning pairs *vs.* other symbol/meaning pairings. The fact that the same pattern of results was observed across two different cultures and languages makes it less likely that they are attributable to a specific cultural or linguistic context.

## 1. Introduction

The notions of archetypes and the collective unconscious, which are central to analytical psychology, have generally remained outside the domain of inquiry of mainstream academic psychology. Nevertheless, there are emerging efforts to integrate ideas from analytical psychology and those drawn from cognitive psychology, neuroscience and even physics, e.g., [1,2,3,4,5,6,7,8,9], *etc.* To date, these efforts have largely aimed at a theoretical or conceptual integration. Attempts to operationalize or empirically test ideas from analytical psychology are still fairly uncommon.

Two studies that did seek to provide an empirical test of the notion of archetypes are therefore noteworthy, see [2,10]. Rosen *et al.* [2] found that participants could not reliably identify the proposed associated meaning of symbols deemed to be archetypal when they relied only on resources available to consciousness. However, when participants were presented with pairs of symbols and meanings to learn in a paired-associate recall procedure, they showed significantly better recall of those pairs in which the archetypal symbols were matched with their associated archetypal meanings than those in which the associated meaning did not correspond to the archetypal meaning. In interpreting their results, the authors theorized that the presentation of the symbol and the associated meaning mobilized prior, implicit associations encoded in memory which under normal conditions are not available to conscious recall. The results of this initial study were subsequently replicated by Huston [11] and Bradshaw and Storm [12].

Although these results may be viewed as lending empirical support to the notion of the existence of collective unconscious (archetypal) memory, they may also reflect linguistic or cultural characteristics of the population tested (native speakers of English in the United States and Australia). To determine whether the obtained effect is not unique to this population it is important to conduct studies with native speakers of other languages, and in other cultural contexts. This was the aim of the present study. In this study we developed a German language adaptation of the materials used by Rosen *et al.* and tested participants residing in the German-speaking part of Switzerland. It was hypothesized that if certain symbols truly have underlying, perhaps universal, “archetypal” meanings, then they should be significantly better recalled if they are paired in a memory task with those meanings than if they are paired with other meanings unrelated to the archetypal ones.

Before proceeding with a description of our study a brief background discussion of archetypes as developed by Jung is in order.

### 1.1. Archetypes

Unlike Freud, Jung believed that the dynamic unconscious was not just the seat of sexual and aggressive instincts and repressed wishes. Through his work with the word association test, the study of myths and fairy tales, and of fantasy products of psychotic patients, Jung reached the conclusion that there was a layer of the unconscious which contains images, patterns of behavior and modes of perception accessible to the whole of the human race (and to the animal world, as well). He named these specific patterns of perception and behavior which crystallize in consciousness in the form of symbols *archetypes* (the word *archetypos* was used by Plato for his ideas and Jung knew this as was pointed out by Barnes [13]). Jung and suggested that archetypes were “empty and purely formal” ([14], p. 79, par. 155), “a possibility of representation given *a priori*” ([14], p. 79, par. 155). Further on, Jung stressed that “the representations themselves are not inherited” ([14], p. 79, par. 155). In this sense, Jung believed that the archetype-as-such is unknowable and “irrepresentable” ([15], p. 213, par. 417); rather, it affects consciousness mainly from its “ability to organize images and ideas” ([15], p. 231, par. 440). In Jung’s view, the archetype “can be named and has an invariable nucleus of meaning—but always only in principle” ([14], p. 80, par. 155). Anything we say about the archetype remains a visualization which is made possible by the current state of consciousness at a given moment. Archetypes for Jung are numinous (that is, highly emotionally charged) and are associated with strong affective responses. Furthermore, the archetype was thought by Jung to have a “psychoid nature” ([15], p. 215, par. 419), which he described as follows: ”the archetype describes a field which exhibits none of the peculiarities of the physiological and yet, in the last analysis, can no longer be regarded as psychic, although it manifests itself psychically” ([15], p. 215, par. 420). In other words, as conceptualized by Jung, archetypes-as-such while being universal are unknowable or unconscious, but can have a profound impact on consciousness and the life of the individual. They do not belong just to the psychic sphere and seem to be given *a priori* as a possibility or as a form without content.

It has been noted that Jung’s account of archetypes is multifaceted. For example, Roesler [9] pointed out that we can speak of at least four different definitions of the archetype in Jung’s writing. The first is a biological definition, according to which the archetype was considered as an inborn pattern of perception and behavior. The second definition is an empirical-statistical one based on Jung’s work with the word association test, according to which the archetype is the nucleus of the categories of complexes noted by him in different individuals. A third definition views archetypes as transcending any particular time, place or individual and whose real nature can never become conscious. Finally, there is a cultural-psychological understanding of the archetype which differentiates between the archetype-as-such and its concrete manifestations which are culturally determined [9]. Although depending on the theoretical orientation there can be significant overlap between these definitions, the research reported here investigates primarily the first, biological, definition of the archetype but it is also compatible with the third definition. 

Contemporary researchers have tried to reformulate the theory of the archetype to make it more compatible with notions in modern science. Among one of the most well formulated approaches is a model which theorizes that what Jung might have meant with the archetype is similar to the contemporary cognitive semanticists’ notion of *image schemas* [3,4,5,16,17,18], that is, a structure of sensorimotor experience that captures a “dynamic, recurring pattern of organism-environment interactions” ([19], p. 136), that can be—“recruited for abstract conceptualization and reasoning” ([19], p. 141). Image schemas are thought to be “preverbal and mostly nonconscious” ([19], p. 144). Jean Knox [3] first proposed a connection between the notion of an image schema and the archetype-as-such. In this sense the archetype is looked at as an early achievement of development resulting from the qualities of the brain as a dynamic system and the interactions between the individual (biological and psychological) and the environment (social, cultural and physical). This understanding of the archetype uses a dynamic systems approach to the development of cognition and action. This approach to cognition and action relates to the process of formation of preverbal image schematic representations in the infant’s brain which are largely determined by the history of the brain as a system, *i.e.*, are based on the experience the system has in the physical world and the ability of the brain as a dynamic system to self-organize [20]. Later on, this pre-verbal neuronal activation pattern serves as a foundation for the development of conceptual thought—categories and concepts. In themselves these neuronal activation patterns constitute attractor states for the dynamic system of the brain.

The idea of the image schema also finds support in contemporary research on embodiment where embodiment is defined as the meaning of symbols to an agent and the reasoning about meaning and sentence understanding which “depends on activity in systems also used for perception, action and emotion” ([21], p. 4). Neuroimaging studies support the idea that sensory and motor systems are involved in concept understanding and retrieval [22]. Thus, image schemas can be understood as neuronal activation patterns which encode embodied experience in the world. They function automatically, *i.e.* unconsciously, and underlie concepts, narrative and ritual [23], all qualities which can be attributed also to archetypes.

Varela, Thomson and Rosch [24] propose a slightly different approach to cognition and action, namely, an *enacted cognition* approach to the study of mental processes and representations. According to this approach, cognition is “enaction: a history of structural coupling that brings forth a world” ([24], p. 172); this view seems consistent with most of the above mentioned ideas. Varela *et al.* go a step further to suggest that “the cognitive system projects its own world, and the apparent reality of this world is merely a reflection of internal laws of the system” ([24], p. 172). 

Among Jungian scholars, George Hogenson [25] looked into the connection between archetypes and mirror neurons and proposed understanding the archetype as an “elementary action pattern” ([25], p. 325), which sounds similar to some of the ideas of the enacted cognition approach of Varela, Thomson and Rosch. Other Jungian scholars stress in their re-interpretation of the nature of the archetype non-linear dynamics which underlie both the functioning of the brain as a system and some aspects of the archetype related to, for example, synchronicity, enantiodromia, or the therapeutic relationship looked at as a dynamic open system. Hogenson proposed that the archetype could be understood as an “iterative moment in the self-organization of the symbolic world” ([26], p. 279). Saunders and Skar have suggested that the archetype is an emergent structure which derives from the self-organizing properties of the brain (a notion very similar to the theory of the image schema) [27]. McDowell stressed that the archetype was a pre-existing principle of the organization of personality [28], while van Eewynk [29,30] looked at archetypes as strange attractors of the dynamic system of the psyche whose non-linear dynamics underlie individuation and the therapeutic relationship. 

Perhaps one of the most controversial aspects of the notion of archetypes is that of innateness. How do we understand innateness and what was actually meant by Jung when he stated that archetypes are *a priori* given to us? Furthermore, how do we understand the innateness of archetypes in an age in which the meanings of symbols are not likely to be transmitted genetically?

While there are still proponents of the idea that archetypes are transmitted genetically (see for further information the review by Roesler [1]), many consider discussions of nature *versus* nurture to be obsolete and stress the *interactionist* nature of human development [1,4,9,17,25,31] or point out psychological factors in evolution in the argumentation against a purely genetically transmitted innateness [32]. The innate aspect of the archetype can also be looked at as predisposition to a genetic condition which needs certain environmental cues to find expression in the sense of epigenetics as described by Roesler [1,9] and Rosen [31,33]. In the light of new discoveries it might well be the case that this epigenetic process which provides the link between environment and genome and determines which genes are being active and which are deactivated might even be more important than the genes themselves and may provide the link between biological substrates—genome and cultural heritage—behavior, habits *etc.* [34]. The Jungian scholar Pietikanen [35] suggested a radical departure from the discussion about innateness and proposed that with the help of a Cassirerian approach archetypes could be understood as “culturally determined functionary forms organizing and structuring certain aspects of man’s cultural activity” ([35], p. 325).

Regarding inborn behavior and archetypes there appears to be empirical support for innateness in experimental psychology for a range of phenomena including the deep structure of language [36], early attachment patterns [37], the idea of “basic emotions”, language acquisition mechanisms, and a face recognition program [1,9]. Roesler [1] points out Seligman’s concept of “preparedness to learn” as a further example of innateness that can be applied to archetypal theory. Similarly, Erik Goodwyn [8,38] uses in defense of innateness findings from evolutionary psychology and neuroanatomy.

We can also say that controversies concerning innateness and the archetype reflect broader controversies in psychology at large. While approaches such as the dynamic systems approach, cognitive semantics, embodiment and enacted cognition as approaches in the study of cognitive processes enjoy widespread popularity, there are also many scholars who conduct experimental work in connection with innate mechanisms. The experimental work of developmental psychologists such as Spelke provides data which supports the hypothesis of multiple innate mechanisms with which infants are equipped at birth. Spelke suggests that “perception, thought, value and action depend on domain-specific cognitive systems” and “each system has its own innate foundations and evolutionary history” ([39], p. 204). For example, in a recent study Izard, Sann, Spelke and Steri [40] report findings that support the assertion that infants at birth are equipped with abstract, numerical representations. Yet other cognitive scientists do not readily accept the notion that there are innate foundations for cognitive capacities, particularly for certain capacities, such as language. It, thus, seems that cognitive science at large is still grappling with questions concerning innateness.

The debate around the nature of the archetype is further enriched by archetypal psychology which sees the place of the archetype in imagination and stresses the transcendental nature of the archetype [1,9]. Although this approach to the archetype might not resonate with many mainstream psychologists, there are tendencies in contemporary studies of consciousness which are compatible with the ideas of archetypal psychology. The Hameroff and Penrose quantum theory of consciousness [41], the idea that consciousness “emerges as natural processes” that involve quantum phenomena “unfold[ing]” [42], and the hypothesis that the brain does not produce consciousness but serves the purpose of receiving and transmitting information which exists from beyond it [43] can all be seen to resonate with some of the basic ideas of archetypal psychology concerning the archetype. Furthermore, the notion of synchronicity—meaningful coincidences—based on an acausal connection principle, which Jung developed in exchange with Wolfgang Pauli and Albert Einstein, and which can be seen as an expression of a constellated archetypal field at work [6,44], finds in recent days, support through discoveries in complexity theory and the dynamics of complex adaptive systems [7]. 

Given all these ideas how are we to understand the archetype? Are archetypes transmitted biologically or are they transmitted by culture as Roesler [1] asks? Can we understand the collective unconscious in terms of subliminal transmission and inter-individual neuronal format as Roesler [1] proposed or is it a form of archetypal memory as Rosen *et al.* [2] suggested? However we reformulate the theory of the archetype and the collective unconscious most Jungian scholars would agree that the basis of the archetype and the collective unconscious is both innate and environmental. The differences are more in terms of degree and the role of each of the two factors.

While the above developments in psychology provide much food for thought, finding a way to test notions about archetypes, however this notion is formulated, would be instructive. We thus turn to two previous empirical studies which attempted such a test and found empirical support in favor of the existence of something akin to archetypes, henceforth termed *the archetype hypothesis*.

### 1.2. Previous Research

Apart from the above mentioned theoretical discussions concerning the nature of the archetype a few scholars have sought to empirically test the hypothesis of archetypes and archetypal memory. As mentioned above, Rosen *et al.* [2], as well as Huston, Rosen and Smith [45], Bradshaw and Storm [12] and Maloney [10] examined this in the domains of memory and preferences. 

Maloney [10] asked a community sample of 151 participants to rate their preferences to images containing archetypal themes and factor analyzed the responses. The images included the archetypal themes of the mother and the hero in both anthropomorphic (e.g., woman gazing lovingly at a child for the positive mother, Hercules for the positive hero) and non-anthropomorphic (e.g., the cave as a symbol of the Great Mother, the heraldic lion as a symbol of the hero) form. Both positive and negative aspects of these themes were examined. The study used an unconstrained Q-sort method. Participants were presented with sets of six images and asked to rate their responses to three questions in respect to the images using a limited set of possible answers. The analysis demonstrated a stable three-factor structure underlying responses to the question “If I were to keep this image with me forever, I would be”. Factor 1 contained images related to a quest theme—the positive hero, the non-anthropomorphic hero, the non-anthropomorphic mother, according to the author. Factor 2 was reported to contain images related to an attachment theme—positive mother. Factor 3 was interpreted as being related to a conflict theme. The author thus concluded that “archetypal structure underlies adult affective responses” ([10], p. 110). Furthermore, Maloney concluded that the images alone were not enough to evoke an archetypal structure, they had to be viewed in a certain way so that the structure was triggered which in the design of his study was achieved through the question that the subjects had to answer. Only the question which required most active participation on the part of the participants in assessing the images yielded significant results.

A different experimental paradigm was developed by Rosen, Smith, Huston and Gonzales [2]. Rosen and colleagues argued that a natural extension of Jung’s own early studies with the Word Association Test would be the study of associations on the basis of symbols. They developed an inventory of forty symbols and forty associated words which were intended to correspond to the symbol’s archetypal meanings—The Archetypal Symbol Inventory (ASI). Furthermore, they designed a cognitive psychological experimental paradigm to test the hypothesis that archetypal symbols were strongly associated to these proposed underlying meanings and that the association lies beyond conscious retrieval under ordinary conditions. Rosen *et al.* conducted a series of three experiments with undergraduate students in psychology at a large university in southwestern U.S. The first two experiments tested participants’ conscious knowledge of the symbols and their meanings. When they were shown each of the ASI symbols, and asked to guess the meaning of each symbol, American participants could not come up with the designated meaning of the symbols. Even more surprisingly, when they were given the 40 ASI symbols with a randomly ordered list of the meanings, participants were unable to match symbols to their correct meanings above the level of chance. These results show that participants were not consciously aware of the meanings of the symbols. The third experiment was a paired-associate learning task in which students (divided into two groups) were first shown all forty symbols. Each group was given half of the symbols matched with the proposed associated meanings and the other half with symbols and meanings mismatched (the particular pairings were counterbalanced across the two groups). After a one minute rest participants were shown only the symbols and were asked to remember and write down the word they initially saw paired with the symbol. It was found that students learned and recalled significantly better the words whose meanings corresponded to the proposed meanings of the archetypal symbols than those that were unrelated to the purported meaning of the symbols. From the list-learning research literature (e.g., [46,47]) it is known that pairs of strongly associated words are learned better than less associated pairs. This gave ground to the authors of the study to conclude that archetypal symbols are strongly associated to the proposed related meanings and that the association is unconscious.

Huston, Rosen and Smith [45] proposed a mechanism to explain the observed effects in the original Rosen *et al.* study and a second variation of the research [11]. They suggested that when a symbol was presented paired with its associated “archetypal” meaning priming occurs which facilitates later recall. The correctly paired symbol with its proposed related meaning also triggers an emotional response which contributes to the “activation and constellation of an archetypal image” ([45], p. 147). The constellated archetypal image and the associated meaning presented to participants together led to priming of memory for the association and facilitated later recall. The mechanism proposed by the above authors is still in the realm of hypothesis and needs to be experimentally tested.

In a recent study Bradshaw and Storm [12] conducted three experiments based on the Rosen and Smith paradigm using 30 out of the original 40 symbols from the ASI in a sample of 237 students and members of the general public in the state of Victoria, Australia. The sample consisted of predominantly Australian/New Zealander citizens (81%) and was predominantly English native speaking (around 86%). The other countries/regions represented were respectively, Britain (3%), Europe (4%), Asia (7%), America (North and South 2%) and Other 3%. The authors replicated the results of Rosen and Smith in the free association task (Experiment 1) and detected in the forced association task (Experiment 2) seven out of 30 symbols which could be consciously known by the participants. For the rest of the symbols there was no statistical evidence in the forced association task for conscious knowledge. The authors modified the paired-associate learning task used in the third experiment of the paradigm. To additionally control for intermediate effects they presented four randomized versions of symbol-word sets, *i.e.* instead of two counterbalancing conditions they had four. Furthermore they modified the timing in the list learning task giving participants 8 seconds in the learning phase as opposed to 5 seconds in the original paradigm and 20 seconds in the recall phase as opposed to 8 seconds in the original paradigm. As stimuli the authors used a set of pictures and drawings of the symbols predominantly downloaded from Internet instead of the original images from the ASI. There was no explanation given for the above modifications. The results replicated the findings of Rosen *et al.* [2] and Huston [11]. Matching words with the symbol that they are associated with, benefitted learning and subsequent recall of the words. The authors reported a statistically significant difference between the different versions of the main experiment. There was a statistically higher recall rate for both matched and mismatched recall in one of the versions. This was partially explained by the age difference between the participants in this version (M = 23 years) and one of the other versions (M = 30 years). No information is available about the mean age in the other groups, as well as the means and standard deviations for matched and mismatched recall in the different groups. Furthermore, the authors detected increased difficulty in learning and recall of mismatched pairs with increased age in their sample (mean age 27, SD = 11 years). No significant interaction between country and ethnicity and performance was found on any of the tasks in all three experiments. This is not surprising since as noted above the sample consisted of predominantly Australian/New Zealander citizens (81%). The number of participants from other countries of origin was very small. As such it could be argued that the sample size of the individual ethnic groups (distributed across the 6 different conditions) was too small to detect any meaningful difference. There is also no information available about how the different ethnic groups or counties of origin were represented across the different experimental conditions. Furthermore, the experiment was carried out in English. All participants, even those who were not native English speakers (14% or less since the authors did not control for language which the participants consider to be their native language) used English as the experimental language. In this sense, it cannot be ruled out that the effect which the authors report (no difference in performance between the different ethnic groups, as well as the significant effect of matching on learning and recall) can be explained by characteristics specific to the English language.

Following its publication the Rosen *et al.* study led others to wonder how robust or generalizable the findings were. Jill Gordon [48] posed the question whether the images used by the team could be considered to be archetypal before additional, cross-cultural, research is conducted using the same paradigm. Similarly, Gordon stressed the importance of conducting cross-cultural studies to determine whether the images used really had the qualities of archetypal images, namely, whether these were “forms that provoke more or less similar or even identical associations from a majority of people” ([48], p. 229). Raya Jones argued in a similar fashion that the results observed by Rosen *et al.* could be explained either in terms of “cultural convention” or as “artifacts of the statistical procedure” ([49], p. 707). 

## 2. Present Study

Motivated by the question of whether the findings of Rosen *et al.* [2] are replicable in a different language and in a different cultural context we decided to conduct the same experiment in another context. We chose for the setting of our study the German-speaking part of Switzerland; although English and German are related languages, there are sufficient cultural differences between the southwestern region of the United States and Switzerland that we felt justified in considering the latter to be a sufficiently different cultural environment. We reasoned that if the results observed by Rosen and colleagues were related to the archetypal nature of the symbols used in the experiments then these results should be replicable in cross-cultural studies conducted in a different language and a different cultural context. 

Thus we hypothesized that if the “archetype hypothesis” has merit, then symbols representing archetypes and their proposed German meanings would also be significantly better learned and recalled than mismatched pairs. The Archetypal Symbol Inventory is composed of forty symbols with occurrence in different cultures and their accepted meanings, that is, the associated accepted meaning of the symbols across cultures. Since the main idea of the present study was to test the replicability of the results from the initial Rosen *et al.* [2] study in a different cultural and linguistic context, it was agreed to apply exactly same procedure for the present experiment.

### 2.1. Participants

A total of 412 college students were recruited for the experiment. They included two different groups of randomly assigned first and second year students from the Medical School at the University of Bern, as well as 14 randomly assigned psychology students from the University of Basel. None of the students had studied archetypal symbolism. Ten students’ data were excluded from the analysis due to incomplete completion of the protocols. Thus the total number of participants in the subsequent analysis was 402. 

The experiment was conducted in two groups (counterbalancing conditions where the participants were assigned randomly). There were 221 students in counterbalancing condition 1 (CB1) and 181 students in counterbalancing condition 2 (CB2). The average age of participants was 21 years; one participant did not indicate her age. Overall 224 women and 178 men took part in the experiment. 

In terms of language background, a total of 366 participants indicated that their primary language was German. An additional 35 participants indicated having a native language other than German; one participant did not indicate native language. The first languages of these participants included Hindi (1), Spanish (2), Serbian (1), French (6), Czech (1), Romansh (3) (the fourth official language of Switzerland), Italian (6), Dutch (1), Tamil (3), Bosnian (1), Croatian (1), Portuguese (1), Turkish (2), Bulgarian (1), English (2), Polish (1), Albanian (1), and Slovenian (1). However, since all participants were studying in fields which required passing a highly competitive entrance exam in German and subsequently took classes and exams in the German language, the research team assumed that the level of language proficiency of these participants is close to that of German speaking native speakers.

### 2.2. Materials

The original English Archetypal Symbol Inventory (ASI) was translated into German (for a description of the process of development of the ASI see Rosen *et al.* [2]). For this purpose the first three authors individually translated the forty items from English to German and then through a process of inter-rater agreement arrived at the final set of German translation equivalents for the forty symbols. An external expert from the Baumann Foundation (Basel) with long experience as a Jungian analyst, supervisor and training analyst, was asked to proof read the translations as well [50].

### 2.3. Procedure

Following Rosen *et al.* [2], a paired-associate learning task was devised. Each group of participants was presented the whole set of forty symbol-word pairs, however, twenty of these were matched with their related meanings and twenty were mismatched, that is, paired with unrelated meanings. The matched pairs in the first counterbalancing condition were presented mismatched in the second counterbalancing condition and *vice versa*. Furthermore, in counterbalancing condition 2 (CB2) the images were shown in reverse order from the order of presentation in counterbalancing condition 1 (CB1) to control for any residual effects related to the order of presentation, as done in the original study. Students were instructed to try to remember the pairs they were shown and received no explanation as to the relationship between the image and the word.

The participants in each group initially saw each image-word pair for 5 seconds and after a 1- minute rest they were shown the images in the same order as in the beginning. This time each image was presented without the word for 8 seconds on the screen. During this time the participants had to try to recall the word they saw initially paired with the image and write it in the respective field of the test protocol. The stimuli were presented using Microsoft Power Point.

Finally, participants were asked to fill out a subjective report consisting of four questions after the end of the experiment. The questions were as follows:

(1) Were any of the image-word pairs familiar to you already before the experiment? If yes, which ones?
(2) Were there among the image-word pairs, ones that you found particularly intriguing? If yes, which ones?
(3) Did you use any particular strategy to be able to learn better the image-word pairs? If yes, then what was it?
(4) Do you have any other comments about the experiment?

## 3. Results

The responses given by participants were scored using a strict criterion. Only words which were the same as the stimulus words or their word forms were coded as “correct”, no synonyms or association words to the stimuli were allowed. Three stimulus words proved to be particularly difficult for the participants—*Unbewusstes* (unconscious), *Vervollständigung* (completion) and *Schöpfungskraft* (generativity). Among the answers there were a small number of word forms such as for *Unbewusstes*—*Unterbewusst(sein)* (34 in CB1 and 14 in CB2), for *Vervollständigung*—*Vollständigkeit* (6 in CB1 and 3 in CB2) and for Schöpfungskraft—*Schöpfung* (57 in CB1 and 35 in CB2) which needed special attention since these were rather distant word forms of the stimulus words. These word forms appeared as answers in both conditions independent of the fact whether the stimulus word was correctly matched with the symbol whose meaning it represents or not. The subsequent analysis demonstrated that the manner of coding of these answers did not affect significantly the results and it was decided to code the word forms as “correct”. 

Furthermore, a technical mistake in the power point presentation of CB2 was discovered. The slide with the mismatched pair-square with the word ‘*Wohltätigkeit’* (charity), had appeared sizably shorter on the screen which had prevented the participants from learning the pair, therefore both symbols affected by the mistake the Square (No. 7) and the Heart (No. 5) were removed from the subsequent analysis in both conditions.

A repeated measures factorial ANOVA with one within-subjects variable (Stimulus Type—matched *vs.* mismatched symbol-meaning pair) and one between subjects variable (Counterbalancing—CB1 *vs.* CB2) was conducted to analyze the data. The means and SD of the recall rates for matched and mismatched pairs in each counterbalancing condition are summarized in Table 1. 

Additionally percentages of correctly recalled matched and mismatched words were calculated for each group following the procedure of Rosen *et al.* [2]. The total number of correctly recalled matched words in each condition was divided by the total possible number of correctly matched responses in the condition and the same procedure was repeated for the mismatched pairs in both conditions. Overall percentages of correctly recalled matched and mismatched words for both conditions were calculated as well. The results are summed in Table 1. In both groups, and for all subjects, the percentage of correctly recalled matched words was higher than the percentage of correctly recalled mismatched words.

**Table 1:** Means, SD and percentage correct answers for both conditions.

	Matched			Mismatched		
	%	Mean	SD	%	Mean	SD
Counterbalance 1 (CB1) (N = 221)	70	12.59	2.66	60.27	12.05	3.29
Counterbalance 2 (CB2) (N = 181)	64.72	12.94	3.3	59.48	10.71	3.2
Total (N = 402)	67.47	12.75	2.97	59.93	11.45	3.32

The main effect of stimulus type was significant, *F* (1, 401) = 125.83, *p* < 0.001, MSE = 3.047, effect size ω^2^ = 0.22; indicating a significantly higher recall accuracy for matched pairs than for mismatched pairs (see Table 1). Matching the symbols with their proposed associated meanings benefited learning and the subsequent recall. 

### 3.1. Item Analysis

We also conducted analysis of the individual items of the ASI following the model of Rosen *et al.* [2]. Our intention was to compare the ranking of the symbols in our study to the ranking which symbols had in the original study. Rosen and team demonstrated that not all symbols were equally useful in their study through calculating an ASI Index for each symbol. The ASI Index was calculated taking into consideration the percentage of correct responses when the symbol and the word were correctly matched and the percentages of correct responses for respectively the symbol and the word when each appeared in a mismatched combination with another word (for the symbol) and another symbol (for the word). For each item the percentage of correct responses when the symbol was mismatched and the percentage correct responses when the word was mismatched were subtracted separately from the percentage correct responses when symbol and word were correctly matched, the two differences were added and divided by two to obtain the ASI index. We conducted the same analysis for all items and the results are presented in Table 2.

**Table 2:** Archetypal Symbol Inventory (ASI) Summary of item analysis: rank-ordered ASI.

Symbol G/E	ASI No.	% correct answers match	% correct answers mismatch symbol	% correct answers mismatch word	ASI Index
Zorn/Wrath	40	96.13	33.03	43.44	57.9
Geburt/Birth	3	97.73	34.81	74.59	43.03
Schönheit/Beauty	2	96.83	41.99	73.48	39.1
Böse/Evil	9	82.81	22.65	70.72	36.13
Rettung/Salvation	29	90.6	62.44	46.61	36.08
Möglichkeit/Possibility	21	72.38	43.44	33.03	34.15
Einheit/Unity	37	74.66	70.72	22.65	27.98
Männlich /Masculine	17	83.43	24.89	88.24	26.87
Macht/Power	23	83.71	54.14	64.64	24.32
Schlaf/Sleep	31	70.59	35.36	58.56	23.63
Schutz/Protection	25	80.54	75.14	40.88	22.53
Leben/Life	16	83.71	65.19	61.88	20.18
Unbewusstes/Unconscious	36	58.56	39.37	48.87	14.44
Gesundheit/Health	14	72.38	67.42	51.13	13.11
Mut/Valor	38	86.74	74.21	74.21	12.53
Geist/Spirit	33	72.4	59.67	60.22	12.46
Potenzial/Potential	22	69.23	64.64	54.14	9.84
Ewigkeit/Eternity	8	63.35	74.59	34.81	8.65
Wissen/Knowledge	15	57.46	42.08	57.92	7.46
Synthese/Synthesis	34	64.09	51.13	67.42	4.82
Aufstieg/Ascent	1	92.27	92.76	83.71	4.04
Reinigung/Purification	26	65.19	66.52	62.44	0.71
Weiblich/Feminine	10	87.85	83.71	92.76	−0.39
Ursprung/Origin	18	62.9	61.88	65.19	−0.64
Perfektion/Perfection	20	52.04	40.88	75.14	−5.97
Rationalität/Rationality	28	40.27	58.56	35.36	−6.69
Zentrum/Center	4	56.91	62.44	66.52	−7.57
Virilität/Virility	39	80.54	89.5	87.29	−7.86
Fruchtbarkeit/Fertility	11	65.75	74.21	74.21	−8.46
Paradox/Paradox	19	64.09	64.25	81.9	−8.99
Seele/Soul	32	64.09	81.9	64.25	−8.99
Schöpfungskraft/Generativity	12	33.7	48.87	39.37	−10.42
Fortschritt/Progress	24	40.33	46.61	62.44	−14.2
Verwandlung/Transformation	35	42.99	60.22	59.67	−16.96
Harmonie/Harmony	13	32.6	57.92	42.08	−17.4
Suche/Quest	27	39.37	73.48	41.99	−18.37
Selbst/Self	30	65.61	87.29	89.5	−22.79
Vervollständigung/Completion	6	9.95	88.24	24.89	−46.62

Items that were recalled better when correctly matched than in any of the other two conditions were ranked the highest. Items that were recalled better when incorrectly matched in both conditions were ranked lowest. 

Although there was a partial overlap of the ranking of items in both the Rosen *et al.* [2] study and our German-speaking study such as having the symbols for power *(Macht)*, unity *(Einheit)*, birth *(Geburt*), masculine *(Männlich)* and protection *(Schutz)* rank among the top third of the ASI index as best recalled when in the matched condition, there were also notable differences. The summary of the comparison of the ranking of the ASI symbols according to their ASI Index for both studies is given in Table 3. Surprisingly symbols as the ones for soul *(Seele)* and feminine *(Weiblich)* dropped to the lowermost third of the ranking in the German study while ranking in the topmost third in the US study. Similarly, the symbol for ascent *(Aufstieg)* that ranked highest in the rank-order of the US ASI study was in the lower end of the middle group of the rank-order in the Swiss study. The ranking of the symbols in the Swiss study was topped by the symbol of wrath *(Zorn)*. 

**Table 3:** Swiss-German ASI Index and US-English ASI Index Comparison.

Symbol G/E	ASI No.	German ASI Index		US ASI Index	
		Ranking	Value	Ranking	Value
Aufstieg/Ascent	1	21	4.04	1	54
Schönheit/Beauty	2	3	39.1	17	11.5
Geburt/Birth	3	2	43.03	8	22
Zentrum/Center	4	27	−7.57	2	47
Vervollständigung/Completion	6	38	−46.62	25	0
Ewigkeit/Eternity	8	18	8.65	19	10.5
Böse/Evil	9	4	36.13	24	1.5
Weiblich/Feminine	10	23	−0.39	10	19.5
Fruchtbarkeit/Fertility	11	29	−8.46	26	0
Schöpfungskraft/Generativity	12	32	−10.42	31	−9
Harmonie/Harmony	13	35	−17.4	30	−8
Gesundheit/Health	14	14	13.11	22	7.5
Wissen/Knowledge	15	19	7.46	20	10
Leben/Life	16	12	20.18	23	7.5
Männlich/Masculine	17	8	26.87	12	15
Ursprung/Origin	18	24	−0.64	34	−15
Paradox/Paradox	19	31	−8.99	11	19
Perfektion/Perfection	20	25	−5.97	14	14
Möglichkeit/Possibility	21	6	34.15	15	14
Potenzial/Potential	22	17	9.84	36	−24.5
Macht/Power	23	9	24.32	5	33
Fortschritt/Progress	24	33	−14.2	27	−4
Schutz/Protection	25	11	22.53	9	20
Reinigung/Purification	26	22	0.71	35	−17.5
Suche/Quest	27	36	−18.37	37	−38
Rationalität/Rationality	28	26	−6.69	33	−11.5
Rettung/Salvation	29	5	36.08	28	−4.5
Selbst/Self	30	37	−22.79	29	−5
Schlaf/Sleep	31	10	23.63	21	9.5
Seele/Soul	32	30	−8.99	4	38
Geist/Spirit	33	16	12.46	18	11
Synthese/Synthesis	34	20	4.82	38	−39
Verwandlung/Transformation	35	34	−16.96	6	33
Unbewusstes/Unconscious	36	13	14.44	32	−11
Einheit/Unity	37	7	27.98	3	46.5
Mut/Valor	38	15	12.53	13	14.5
Virilität/Virility	39	28	−7.86	7	33
Zorn/Wrath	40	1	57.9	16	12

The observed differences can possibly be explained by the different contexts of the samples in the two studies, *i.e.*, socio-cultural factors might have exerted an influence on the results. These may include, for example, cultural value systems, cultural complexes, and/or current culturally specific social, economic and political issues. Central themes for the participants at the time of the experiment might have also affected the results (e.g., the nearing of exam session for the medical students). Among the psycholinguistic factors that could have affected the observed results are word length and frequency of use in daily speech for the respective word-stimuli used in the experiment. As stated earlier some of the verbal stimuli in German presented a significant challenge for the participants (e.g., *Unbewusstes* (unconscious), *Vervollständigung* (completion) and *Schöpfungskraft* (generativity)).

### 3.2. Subjective Report

A total of 184 out of 221 participants in CB1 and 108 out of 181 participants in CB2 indicated that they did not know any of the image-word pairs used in the experiment before taking part in it. Among the rest of the participants in both groups there were participants who listed some pairs—both matched and mismatched—as already familiar.

In CB 1 the pairs that were listed by the highest number of people as familiar were *Taube—Geist* (pigeon—spirit) named by 14 participants and *Ring—Ewigkeit* (ring—eternity) written by 8 participants. This is not surprising since both pairs are culturally well-known. The participants in CB2 listed as familiar the combinations *Schlange—Gesundheit/ Medizin* (snake—health/medicine) named by 29 people, *Treppe—Aufstieg* (stairs—ascent) named by 18 participants, *Mond—Weiblich* (moon—feminine) listed by 12 people, *Sonne—Männlich* (sun—masculine) written by 11 people, *Arche—Rettung* (ark—salvation) named by 9 participants and *Apfel – Wissen* (apple—knowledge) written by 6 participants. In this case as well, most of the symbols, listed as familiar from before the experiment, are well culturally known symbols. We can also say that the association between the snake and health/medicine is related to the major of the participants in our study (medicine).

To control for previous conscious knowledge of the above pairs listed by the participants in their subjective report, we identified and excluded from the analysis all correct answers which corresponded to the pairs listed by the respective participants as familiar from before the experiment. The data were then reanalyzed. There was no change in the results. The effect of matching on learning and recall was still significant, *F* (1, 401) = 55.78, *p* < 0.001. Thus we can say that even after controlling for previous knowledge the appropriate matching of the symbols with the associated meaning benefited learning and subsequent recall of the words and the associations were not considered to be consciously familiar by the participants.

Almost all pairs—both matched and mismatched—in both groups were listed by some participants as intriguing. Some participants indicated that the intriguing pairs were the ones that they listed as familiar. These answers are particularly interesting since they raise the question about the subjective experience of the participants during the experiment and the personal associations of participants. While this was outside the scope of the present study it is worthwhile investigating in subsequent studies.

A total of 41 participants in CB1 and 12 participants in CB 2 answered that they used no strategy in learning the pairs in the experiment. However, many participants listed a number of strategies they used to learn better the image-word pairs. Among these the most common ones were: making associations between image and word, mentioned by 71 participants in CB1 and 48 in CB2, constructing stories/sentences with the image and the word, named by 61 participants in CB1 and by 74 participants in CB2, building associations to previous experiences or known facts, given by 23 participants in CB1 and 18 in CB2, finding a personal meaning or associating to a personal memory (memory aid) by 12 people in CB1 and 14 in CB2, connecting image and word with emotions , named by 2 people in CB1 and 5 in CB2, constructing scenes or pictures with the image and the word, listed by 13 people in CB1 and 9 in CB2. It is of particular interest that participants note the use of personal experience or associations related to the image-word pairs, as well as emotion. The last strategy relates to the mechanism proposed by Huston *et al.* [45] which explains the observed effect of matching where the constellated archetypal image evokes an affective response and the affect facilitates the later recall of the word through building association with personal experiences. However, these subjective reports do not suffice as proof of the mechanisms and further research is necessary before any definite statements can be made.

Among the more common remarks about the experiment were suggestions for improvement of the experimental design such as including numbers on the slides with the images in the second part, showing the image-word pairs longer on the screen, reducing the number of images. Some included comments concerning the fit of image and word (these did not fit together) or mentioned being able to recall the associations but not the words. These remarks are not surprising and demonstrate the difficulty which the experiment presented for the participants.

## 4. Discussion

The cross-cultural study of the associations between archetypal symbols and their proposed meanings in a German-speaking sample of Swiss students replicated the findings of Rosen *et al.* [2] and demonstrated that there was a highly significant effect of matching on learning and subsequent recall of words correctly matched with the archetypal symbols whose meaning they represent. These results extend to Swiss German speakers the findings of Rosen and colleagues [2] reported in a sample of English speaking students. Being able to replicate the findings of superior memory for related than unrelated pairs in a German speaking sample provides further evidence that archetypal symbols are truly associated with their accepted meanings. The fact that even after excluding the pairs which were listed by the participants as familiar from before the experiment the effect of matching on learning and recall was still highly significant supports the hypothesis that the associations between symbols and their meanings are not conscious. Furthermore, this cross-cultural evidence of the association between archetypal symbols and their meanings demonstrates that it is less likely that the observed effect is related to cultural context or is a linguistic artifact. In this sense, it can be said that our results provide more evidence that the collective unconscious and archetypes as hypothesized by C. G. Jung might have a universal nature. 

The differences in the rank—order of the archetypal symbols in the US study and in the Swiss-German study suggest that it is likely that depending on circumstances some archetypes come to the fore and affect stronger conscious life than others. As mentioned earlier, according to Jungian scholars, we all have the potential or predisposition to recognize the archetypal image, however, our environment influences our experiences. The differences in the rank-order of the items in both the US and the Swiss-German ASI studies empirically support such reasoning. It is highly interesting that some symbols which at first glance seem to have an obvious association to their proposed meaning were not ranked high as would be expected—e.g., Ascent *(Aufstieg)*. Also symbols that were highly culturally bound such as the symbol for soul *(Seele*), for example, dropped in the lowermost third of the ranking against our expectations. Since we do not know how exactly the symbol-word pairs represent the archetypes and how the archetype enhances memory, as Bradshaw and Storm [12] point out as well, the index and the comparison between the different studies can potentially hint to processes which are at work. It might well be that this Symbol Association Test which Rosen and Smith first proposed functions similar to the Word Association Test used by Jung, in the work with which Jung first came across the phenomenon of the archetype. More research is needed on the personal associations of participants involved in the paired associate task and cross-sample comparison of the indexes for each item to be able to make definite conclusions.

Furthermore, some participants indicated in their subjective report that there were pairs they knew from before the experiment. It is of course possible that the participants were familiar with the indicated pairs, since most of the pairs mentioned as familiar were culturally known symbols. However, it is also noteworthy that this was an experiment where archetypal associations were investigated and it is known that often an archetypal experience, correlating the presentation of an archetypal image and meaning, is followed by a strong feeling of having already known the experience or familiarity [51]. Regardless it is clear that among the pairs listed as familiar there were some mismatched pairs. While from a Jungian point of view this must indicate strong personal associations reflecting the activation of a complex, it would also be interesting to research this phenomenon in the context of illusions of competence in monitoring one’s won knowledge as done by Koriat and Bjork [46].

Although our empirical investigation demonstrated that archetypal symbols are strongly associated in two different cultures and two different languages, English and German are languages from the same language group and share many similarities. Therefore, to convincingly demonstrate the universality of these findings, future research should attempt to replicate the experiment in non-Indo-European languages such as Japanese, Chinese, Turkish, Hebrew, Arabic, *etc.* or other Indo-European languages which are less related to English and German, such as Slavic languages for example. Furthermore, it would be of interest to conduct the paired associate learning task with the archetypal symbols from the ASI and their associated meanings in a larger sample of bilingual participants to test if bilingual participants will demonstrate the same pattern of learning and recall.

Although the cross-cultural replication of the original study with the Archetypal Symbol Inventory replicated the findings, there still are many questions that deserve further research. A question raised by a reviewer of this article and addressed by Bradshaw and Storm [12] is whether the observed significant effect of matching cannot simply be explained by the fact that meaning-words demonstrate a degree of descriptive similarity to the visual images of the symbols from the ASI. To control for a possible effect of descriptive similarity between the image and its associated meaning-word on the observed results, symbols and meaning-words were presented also mismatched to the participants. As already noted by Rosen *et al.* [2] some words were better learned and recalled when mismatched as reflected in the calculation of the index in item analyses. A similar phenomenon was observed by Bradshaw and Storm [12] as well. These authors reported having identified six words which were recalled better when mismatched. They argued that this memory enhancement could be based on descriptive similarity. The question is whether this phenomenon is not better explained as resulting from the personal associations of the participants and the complexes which were triggered rather than descriptive similarity. The very fact that there is such variability of learning and recall of the words from the ASI in the different samples as demonstrated by the comparison of the indexes in the item analyses of the US study and the Swiss study would seem to support such a hypothesis. However, further research on the associations of people using symbols from the ASI is necessary to be able to have a better understanding of the processes involved.

Furthermore, whereas there is clearly a strong association between the archetypal symbols and their proposed meanings independent of linguistic and cultural context, it still is not exactly clear how this can be explained. Are the observed results due to the effect of embodiment on cognition in terms of the dynamic system’s approach to cognition and action and the theory of image schema? The embodied cognition approach proposes that “cognitive processes are deeply rooted in the body’s interactions with the world” ([52], p. 625). What is more, this approach argues that “we represent our knowledge together with the sensory and motor features that were activated during its acquisition” ([53], p. 161), and which in part constitute the image schemas as neuronal activation patterns that underlie even abstract knowledge and concepts [19]. As pointed out earlier, the dynamic systems approach to the development of cognition and action suggests that as a result of experience attractor states are formed in the infant’s brain; these correspond to particular neuronal activation patterns which encode the experience resulting from the interaction of the organism and the environment where the environment has to be understood both as social and physical. These patterns underlie also conceptual understanding and are associated with feelings which have accompanied the respective experience. These basic patterns of neuronal activation form the basis of most of our cognitive and emotional functioning. In this sense it seems worthwhile experimentally investigating the hypothesis that the associations between archetypal symbols and their meanings can be explained in terms of encoding the same sensory-motor experience in a different form. Testing this hypothesis experimentally can also provide evidence in favor of or against the assertions that the archetype-as-such can be understood in terms of image schema.

Do our results, on the other hand, support the debated innateness of the archetype? Although our study found out that in different language and cultural contexts archetypal (presumably universal) symbols are strongly associated to their accepted meanings and the nature of this association is unconscious, the question still remains whether this memory effect can be explained as a result of innate mechanisms and predispositions or as Roesler points out using Seligman’s term “preparedness to learn” as an innate factor, or if the observed memory effect can be viewed as resulting from the quality of the brain as a system to form stable attractor states based on accumulated experience in the environment both physical and social (image schemas). We could demonstrate the presence of unconscious implicit memory of the associations between symbols and their proposed meanings in the absence of conscious awareness of the associations, but the source and quality of this form of memory needs further investigation. It would be particularly interesting to conduct functional brain imaging of participants involved in the main experiment to be able to delineate the activation pattern which underlies the performance on the cognitive tasks involved in the main study. Furthermore, comparing the activation pattern observed in such a study to the activation pattern underlying a constellated complex from the brain imaging study of Bechtel [54] could shed more light as to the neural correlates underlying the complex and the archetype.

Although we could demonstrate that participants from two different language and cultural backgrounds could more easily learn and recall matched archetypal symbol-meaning pairs, the question remains whether these associations are moderated by age. Bradshaw and Storm [12] demonstrated a significant correlation between age and learning and recall of mismatched pairs in a sample of 154 participants with mean age of 27 years (SD = 11 years). However, the question still remains whether the results are replicable among the elderly and/or children. Demonstrating that in a large enough sample of children or elderly presenting the symbols together with their archetypal meanings benefits learning and subsequent recall of words would be a further argument supporting the proposed by Jung universality of the archetype and is a necessary further step in this line of research. Furthermore, it would be of interest to conduct the experiment with patients who have amnesia, as suggested by Huston, Rosen and Smith [45]. Results from such a study would be revealing as to the type of memory involved in the mechanisms which underlie the observed effects.

Given the answers of the participants to the questions in the subjective report it seems also worthwhile to investigate the subjective experience of the participants when they are presented the symbol-word pairs and in this sense to systematically use symbols in the study of personal associations in a manner similar to the studies conducted using the Word Association Test. Thus developing a symbols association test would be a further important step in the study of the complex and the archetype.

Furthermore, all the studies based on the Rosen and Smith paradigm until now were conducted in samples of students or the general public. In this sense, it would be interesting to conduct studies using the ASI with Jungian analysts. It would also be particularly valuable to test the model validity of the ASI in a study with trained Jungian analysts and or Jungian scholars to test the degree to which this model of presentation of the archetype is acceptable to the general Jungian community.

Finally, it is important to acknowledge that although our findings are consistent with the framework of archetypes that there may be other underlying factors that may have made the matched pairs easier to learn and recall than the mismatched pairs. Possible stimulus-related characteristics to screen in additional research would be word length and frequency of daily use for the verbal stimuli. 

## 5. Conclusion

To conclude, our study demonstrated that presenting symbols matched with their accepted meanings exerts a statistically significant effect on learning and recall independent of language and culture, even though participants lack conscious awareness of the associations. Our findings which replicated the initial findings of Rosen *et al.* [2], suggest that there is indeed an “archetypal memory advantage”. However, there is need for further experimental work to be able to answer many of the questions concerning the nature of the archetype and the collective unconscious.
